# pH‐dependent electrochemical oxidation of 5‐hydroxymethylfurfural: Reaction mechanism, catalyst design, and reactor design across alkaline to acidic media

**DOI:** 10.1002/smo2.70027

**Published:** 2025-11-24

**Authors:** Peiyun Zhou, Xikang Zhao, Yang Song, Ruixiang Ge, Haohong Duan

**Affiliations:** ^1^ Department of Chemistry Tsinghua University Beijing China; ^2^ Research Center for Renewable Energy SINOPEC Research Institute of Petroleum Processing Beijing China; ^3^ State Key Laboratory of Petroleum Molecular and Process Engineering Beijing China; ^4^ College of Chemical and Biological Engineering Shandong University of Science and Technology Qingdao China

**Keywords:** biomass valorization, electrocatalyst design, full pH, HMF electrooxidation, reaction mechanism, reactor design

## Abstract

The electrochemical oxidation of biomass‐derived platform molecule 5‐hydroxymethylfurfural (HMF) represents a crucial pathway for green transformation into high‐value chemicals, yet its reaction pathway selectivity, efficiency, and catalyst stability are strongly dependent on the electrolyte pH environment. Under alkaline conditions, high OH^−^ concentration facilitates preferential aldehyde group oxidation and efficient deprotonation, enabling highly efficient synthesis of 2,5‐furandicarboxylic acid, but simultaneously induces HMF self‐degradation and complicates product separation. As pH decreases, the reaction mechanism shifts toward enhanced hydroxymethyl oxidation, leading to intermediate accumulation (such as 5‐hydroxymethyl‐2‐furancarboxylic acid, 2,5‐diformylfuran, and 5‐formyl‐2‐furancarboxylic acid) with challenging selectivity control and significantly slowed reaction kinetics. This review comprehensively examines the systematic differences in HMF oxidation pathways and surface catalytic mechanisms across the full pH range from alkaline to acidic conditions. Addressing the distinct reaction characteristics and core challenges in alkaline, near‐neutral, and acidic media, we systematically evaluate design strategies for high‐efficiency electrocatalysts and explore reactor design aspects. Future research should focus on process integration (with tailored reactor design) for energy consumption reduction in alkaline systems, targeted synthesis of diverse oxidation products in near‐neutral systems, and innovative catalyst development for acidic systems, thereby advancing the efficiency, selectivity, and practical application of HMF electrooxidation technologies across the entire pH spectrum through synergistic optimization of catalyst, reactor, and process.

## Graphical abstract

This review summarizes the progress in pH‐dependent electrochemical oxidation of 5‐hydroxymethylfurfural for value‐added chemical production. It highlights how pH dictates reaction mechanisms and product selectivity, discusses strategies for designing effective electrocatalysts tailored for alkaline, near‐neutral, or acidic electrolytes, and explores advanced reactor design for potential industrial applications.

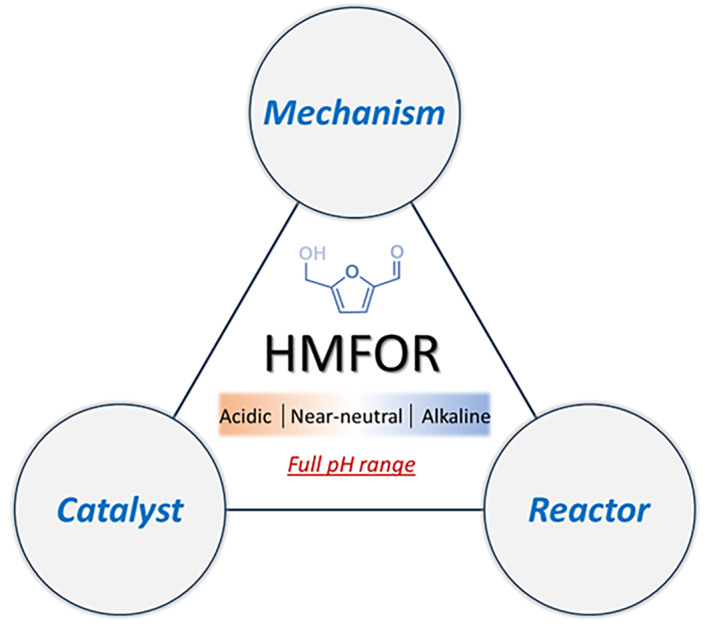

## 1 INTRODUCTION

Biomass, owing to its net zero carbon emission profile, is emerging as an ideal substitute for increasingly scarce traditional fossil fuels.[^1^, ^2^] The efficient conversion of biomass‐derived platform molecules into high‐value‐added chemicals is pivotal for advancing sustainable chemical production.^[3]^ Among these, 5‐hydroxymethylfurfural (HMF), a key platform molecule efficiently derivable from cellulose and hemicellulose, holds a central position.^[4]^ The unique aldehyde, hydroxymethyl, and furan ring functionalities within the HMF molecule enable its transformation into diverse high‐value‐added chemicals via oxidation pathways with varying degrees of oxidation.^[5]^ These products include 5‐hydroxymethyl‐2‐furancarboxylic acid (HMFCA), 2,5‐diformylfuran (DFF), 5‐formyl‐2‐furancarboxylic acid (FFCA), 2,5‐furandicarboxylic acid (FDCA), and maleic acid (MA).[^5^, ^6^] These compounds find extensive applications: HMFCA serves as a crucial monomer for polyester synthesis; FFCA exhibits significant potential in fuels, chemical intermediates, and pharmaceuticals; DFF is utilized in synthesizing pharmaceutical intermediates, diamines, antifungal agents, polyols, and polyethylene materials; while MA is a key raw material for producing lubricant additives and agricultural chemicals.[^5^, ^7^] Notably, FDCA represents a promising bio‐based alternative to petroleum‐derived terephthalic acid (TPA) for manufacturing polyethylene furandicarboxylate plastics, potentially displacing conventional polyethylene terephthalate plastics with an annual production exceeding 70 million tons.^[8]^

Recently, multiple catalytic oxidation pathways have been developed for upgrading HMF, primarily including thermal catalysis, photocatalysis, and electrocatalysis.[^9^, ^10^, ^11^] Thermal catalysis, while capable of achieving high reaction rates, typically relies on chemical oxidants or pressurized O_2_ and operates under harsh conditions (temperatures ≥50°C and pressures ≥10 bar), leading to concerns regarding energy consumption and sustainability.^[9]^ Photocatalysis harnesses solar energy as a renewable driving force, offering great potential; however, it often faces challenges such as limited efficiency due to rapid charge carrier recombination and sluggish reaction kinetics.[^11^, ^12^] In contrast, electrochemical oxidation (ECO) technology has emerged as an attractive pathway for upgrading HMF to high‐value‐added products.^[10]^ Through the utilization of renewable electricity as the driving force and water as the oxygen source under ambient temperature and pressure, the ECO approach demonstrates superior green and sustainable potential.[^13^, ^14^]

**FIGURE 1:**
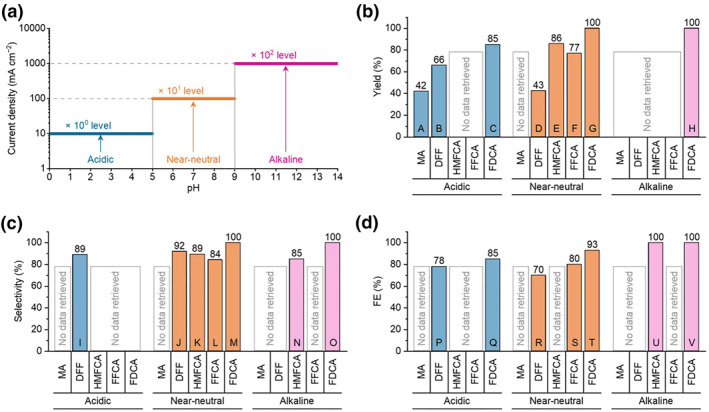
(a) The relationship between the order of magnitude of HMFOR current density and solution pH in most reported articles (Table 1). The highest (b) yield, (c) selectivity, and (d) FE values reported so far. Refs.: A,^[82]^ B,^[84]^ C,^[84]^ D,^[17]^ E,^[79]^ F,^[76]^ G,^[71]^ H,[^29^, ^51^, ^58^] I,^[80]^ J,^[77]^ K,^[79]^ L,^[76]^ M,^[71]^ N,^[64]^ O,[^30^, ^35^, ^49^, ^63^] P,^[84]^ Q,^[84]^ R,^[17]^ S,^[70]^ T,^[68]^ U,^[64]^ V.[^43^, ^51^, ^53^, ^58^, ^64^] FE, Faradaic efficiency; HMFOR, 5‐hydroxymethylfurfural oxidation reaction.

However, a critical and pervasive challenge hindering the practical application of ECO is its pronounced pH dependence: reaction performance—encompassing activity, selectivity, reaction pathways, and catalyst stability—exhibits high sensitivity to electrolyte pH.[^5^, ^6^, ^15^, ^16^, ^17^] To date, literature reports on efficient HMF‐to‐FDCA conversion predominantly occur in strong alkaline media (pH ≥ 13) (Table 1). Under these conditions, abundant OH^−^ ions facilitate key steps, such as nucleophilic attack on the aldehyde group and efficient deprotonation of intermediates, enabling high FDCA yields (>90%) on noble metals (e.g., Ir, Pt, Ru)‐based or transition metals (e.g., Ni, Co, Cu)‐based catalysts.[^17^, ^22^, ^25^, ^31^, ^46^] Nonetheless, reliance on strong alkaline media introduces significant drawbacks: (1) HMF instability under high alkalinity promotes base‐catalyzed self‐degradation or polymerization, increasing carbon loss[^16^, ^62^]; (2) alkaline electrolytes complicate downstream product separation (e.g., FDCA precipitation requires acidic conditions) and electrolyte regeneration, incurring substantial energy and economic costs[^33^, ^85^]; (3) numerous practical applications necessitate operation under near‐neutral (pH 5–9) or even acidic (pH ≤ 5) conditions—such as utilizing acidic biomass hydrolysates directly or compatibility with specific electrochemical reactor configurations employing proton exchange membranes.[^86^, ^87^] Unfortunately, electrochemical HMF oxidation reaction (HMFOR) performance deteriorates markedly under near‐neutral/acidic conditions. This manifests most prominently as sluggish reaction kinetics, typically resulting in current densities one to two orders of magnitude lower than under alkaline conditions (Figure 1a). Furthermore, electrolyte pH profoundly influences the product distribution: strong alkalinity favors preferential oxidation of the aldehyde group over the hydroxymethyl group, while non‐strongly alkaline conditions often promote oxidation of the hydroxymethyl group.[^2^, ^16^] The reported optimal yields, selectivities, and Faradaic efficiencies (FEs) (Figure 1b–d) illustrate this dependency clearly: alkaline media primarily produce FDCA with 100% yield, selectivity, and FE. As pH decreases, the product distribution becomes increasingly complex, stemming from significant pH‐induced alterations in the oxidation pathways and kinetics of the aldehyde and hydroxymethyl groups, impacting overall oxidation degree and selectivity.

**TABLE 1:** Summary of representative catalyst performance under different pH environments.

Medium	Electrolyte (pH)	Material	HMF initial concentration (mM)	j(mA cm^−2^)@E(V_RHE_) in LSV curve	Main product	FE (%)	Sel. (%)	Yield (%)	Refs
Strong alkaline	1 M KOH (pH 14)	Co_4_N@CeO_2_	300	100@1.32	FDCA	86.5	84.7	NA	[18]
				200@1.36					
	1 M KOH (pH 14)	CuO‐PdO	50	∼12@1.35	FDCA	93.7	NA	96.2	[19]
	1 M KOH (pH 14)	NiS_*x*_/β‐Ni(OH)_2_/Ni	50	∼60@1.35	FDCA	98.3	97.7	NA	[20]
	1 M KOH (pH 14)	NiCo_2_@MoO_2_/NF	10	10@1.20	FDCA	99.0	99.2	∼98.6	[21]
				100@1.271					
	1 M KOH (pH 14)	NF@Co_3_O_4_/CeO_2_	50	10@1.33	FDCA	97.5	NA	94.5	[22]
				∼32@1.40					
	1 M KOH (pH 14)	CuCo‐V_2_O_3_	10	10@1.27	FDCA	98.8	99.1	NA	[23]
				50@1.30					
				100@1.32					
	1 M KOH (pH 14)	CuO/Co_3_O_4_	50	10@1.45	FDCA	88	NA	81	[24]
				∼15@1.50					
	1 M KOH (pH 14)	Pt/CuO@CF	10	119@1.50	FDCA	99	99	99	[25]
	1 M KOH (pH 14)	NF/CoOOH@CeO_2_	50	50@1.29	FDCA	98.5	92.5	NA	[26]
				103.9@1.40					
	1 M KOH (pH 14)	R‐ZIF‐CoNi(g)	10	10@1.36	FDCA	98.5	NA	99.6	[27]
				∼80@1.50					
	1 M KOH (pH 14)	Ni(OH)_2_‐NiOOH/NiFeP	10	∼70@1.40	FDCA	94.62	NA	99.4	[28]
				∼110@1.45					
	1 M KOH (pH 14)	NiMo_3_S_4_‐R	10	10@1.336	FDCA	98.5	98.7	∼100	[29]
				100@1.395					
				∼250@1.50					
	1 M KOH (pH 14)	NiCoB_*x*_/NF	10	10@1.27	FDCA	95	100	99.7	[30]
				126@1.47					
	1 M KOH (pH 14)	Ir‐Co_3_O_4_	50	∼11@1.40	FDCA	98	NA	98	[31]
	1 M KOH (pH 14)	Rh‐SA/NiFe NMLDH	50	50@1.30	FDCA	98.5	99.8	98	[32]
				∼95@1.40					
	0.1 M KOH (pH 13)	Ru_1_‐Co_3_O_4_	50	10@1.191	FDCA	86.8	NA	99.3	[33]
				∼36@1.40					
	1 M KOH (pH 14)	Ni_0.5_Co_2.5_O_4_	50	∼15@1.40	FDCA	90.35	NA	92.42	[34]
	1 M KOH (pH 14)	Co_0.4_NiS@NF	50	244@1.35	FDCA	99.1	∼100	NA	[35]
				368@1.40					
				497@1.45					
				50@1.24					
				100@1.29					
				200@1.34					
	1 M KOH (pH 14)	Mn_0.2_NiS/GF	100	100@1.35	FDCA	94.2	98.3	97.6	[36]
				500@1.48					
	1 M KOH (pH 14)	Amorphous Mn‐doped NiFeB alloy	5	∼50@1.40	FDCA	94.2	88	86.6	[37]
	1 M KOH (pH 14)	Cr‐Ni(OH)_2_	10	230@1.47	FDCA	96	95.5	NA	[38]
	1 M KOH (pH 14)	NiO‐Co_3_O_4_	10	50@1.37	FDCA	96	NA	98	[39]
				∼55@1.40					
	1 M KOH (pH 14)	E‐CoAl‐LDH‐NSA	10	10@1.30	FDCA	99.4	NA	NA	[40]
				100@1.59					
	1 M KOH (pH 14)	d‐NiFe LDH/CP	10	∼16@1.50	FDCA	84.47	NA	96.8	[41]
	1 M KOH (pH 14)	F‐doped NiCo_2_O_4_	50	14.4@1.40	FDCA	96.5	97	NA	[42]
				26.5@1.45					
	1 M KOH (pH 14)	NiVW_*v*_‐LMH	10	193@1.43	FDCA	∼100	99.2	NA	[43]
	1 M KOH (pH 14)	S─O_v_─LDH	50	10@1.26	FDCA	94.9	NA	98.4	[44]
				50@1.39					
				∼100@1.50					
	1 M KOH (pH 14)	Pt‐Vco	50	∼7@1.35	FDCA	95.8	NA	97.3	[45]
				∼30@1.50					
	1 M KOH (pH 14)	CuCo_2_O_4_	50	150@1.37	FDCA	94	NA	93.7	[46]
				∼155@1.40					
	1 M KOH (pH 14)	NiCo_2_O_4_	10	50@1.47	FDCA	>99	>99	>99	[47]
				30@1.34					
				∼52@1.50					
				∼35@1.40					
	1 M KOH (pH 14)	NiO(111)	20	10@1.39	FDCA	98.6	∼99	>97	[48]
	1 M KOH (pH 14)	CoCu	50	16.71@1.38	FDCA	96	∼100	96.2	[49]
	1 M KOH (pH 14)	Pt/Ni(OH)_2_	50	37.31@1.50	FDCA	98.7	NA	NA	[50]
	1 M KOH (pH 14)	Pt_26_Ni_74_	50	∼270@1.40	FDCA	∼100	NA	∼100	[51]
				∼330@1.45					
				∼350@1.50					
	0.1 M KOH (pH 13)	NiCoBDC	10	∼3@1.50	FDCA	78.8	95	99	[52]
	1 M KOH (pH 14)	CoNiFe‐MOFs/NF	10	100@1.35	FDCA	∼100	NA	99.76	[53]
				∼125@1.40					
	10 mM KOH (pH 12)	Ni‐MOF‐74D	10	NA	FDCA	80	NA	95	[54]
	0.1 M KOH (pH 13)	Ni‐HITP/PW_12_	10	10@1.25	FDCA	98	NA	99	[55]
				100@1.35					
				∼230@1.40					
	1 M KOH (pH 14)	t‐Ni1Co1‐MOF	50	56@1.30	FDCA	98	NA	NA	[56]
				315@1.35					
				600@1.40					
	1 M KOH (pH 14)	Ni−Cu/NF	50	1000@1.50	FDCA	99.7	NA	99.5	[57]
	1 M KOH (pH 14)	CC@CoNiMnCuZnCdMg‐LHA	20	100@1.42	FDCA	∼100	NA	∼100	[58]
	1 M KOH (pH 14)	BZ‐NiCo(OH)_*x*_	50	111.2@1.40	FDCA	95.39	99.17	95.24	[59]
				100@1.38					
	1 M KOH (pH 14)	Ni(OH)_2_–TPA	100	674@1.50	FDCA	96.8	NA	97.3	[60]
	1 M KOH (pH 14)	NiC_2_O_4_/NF	10	∼55@1.40	FDCA	97.8	NA	97.1	[61]
	1 M KOH (pH 14)	CoOOH/NF	600	NA	FDCA	∼80	96.9	NA	[62]
	1 M KOH (pH 14)	NiCuO_*x*_/NF	100	300@1.36	FDCA	99.5	∼100	99.5	[63]
				500@1.37					
				1000@1.42					
	1 M KOH (pH 14)	Au‐Ni	50	∼20@0.57 (CV)	HMFCA	∼100	85	NA	[64]
	1 M KOH (pH 14)	A‐Co‐Ni_2_P	100	362@1.40	FDCA	NA	NA	99.2	[65]
				583@1.45					
				812@1.50					
				1290@1.60					
Weak alkaline	1 M carbonate buffer (pH 11)	CoO_*x*_‐CrO_*x*_	100	50.1@1.5	FDCA	90	92	89	[16]
	0.5 M sodium borate buffer solution with 8 mM TEMPO (pH 10)	Carbon felt	20	∼3.2@1.2	FDCA	96.7	NA	96.7	[66]
	0.5 M sodium borate buffer solution with 8 mM ACT (pH 10)	Carbon felt	20	∼2.8@1.3	FDCA	93.5	NA	93.5	[66]
	0.5 M borate buffer solution with 7.5 mM TEMPO (pH 9.2)	Carbon‐felt	5	∼1@1.1	FDCA	93	NA	99	[67]
Near‐neutral	1 M phosphate buffer solution with 50 mM TEMPO (pH 7)	Co_3_O_4_	100	14@1.55	FDCA	93	NA	99	[68]
	1 M phosphate buffer solution (pH 7)	S‐Ru/MnO_2_	50	47@0.95–1.55 (pulse electrolysis)	FDCA	79	NA	98.7	[69]
	1 M KHCO_3_ (pH 8.31)	pCoHA‐Ru	100	100@1.53	FFCA	>80	84.3	NA	[70]
				65.7@1.45					
				135.5@1.45 (60°C)	FDCA	NA	∼90.0 (two‐step electrolysis, 60°C)	∼92.1 (two‐step electrolysis, 60°C)	[70]
	Decoupling strategy: 1 M KOH (pH ∼14), pure water (pH ∼7)	Ni_0.85_Co_0.15_(OH)_2_	5	NA	FDCA	79.7 (40°C)	∼100	∼100	[71]
	CO_2_‐saturated 0.5 M KHCO_3_ (pH 7.2)	NiO NPs	10	∼0.6@1.5	FFCA	∼40	NA	>60	[72]
				∼3.4@1.6					
	1 M phosphate buffer solution (pH ∼7)	Vo‐Co_3_O_4_	10	10@1.53	FFCA	NA	NA	∼60	[73]
	0.2 M phosphate buffer solution (pH 7.4)	V‐NiO‐Rh	10	10@1.63	FFCA	NA	71	51	[74]
				∼4@1.5					
	0.5 M KHCO_3_ (pH 7.2)	Co_3_O_4_/CuPc	50	∼2@1.5	FFCA	∼60	NA	64	[75]
	0.2 M phosphate buffer solution (pH 7.4)	NiO_*x*_‐PtO_*x*_/CF	10	10@1.83	FFCA	NA	84	77	[76]
				∼5@1.5					
	0.1 M Na_2_B_4_O_7_ (pH 7.0)	Co_8_Ce_2_O_*x*_	5	∼0.2@1.60	DFF	NA	92	NA	[77]
	1.0 M phosphate buffer solution (pH ∼7)	Ru_1_‐NiO	50	10@1.283	DFF	70	90	42.5	[17]
				∼30@1.5					
	0.5 M HAc/NaAc buffer solution (pH 6)	Ru_4_/PEI‐rGO	5	∼8@1.5	DFF	51.7	66.2	NA	[78]
	0.1 M phosphate buffer solution (pH 7)	Co(OH)_2_‐CeO_2_	10	32@1.5	HMFCA	NA	89.4	85.8	[79]
Acidic	100 mM H_2_SO_4_ (pH∼1)	PtRu(1:1)	100	NA	DFF	NA	89 (50°C)	22 (50°C)	[80]
	H_2_SO_4_ (pH 1)	MnO_*x*_	20	∼1.5@1.6	FDCA	33.8 (60°C)	NA	53.8 (60°C)	[6]
				∼1@1.5	MA	NA	NA	21.9 (60°C)	[6]
	0.1 M HClO_4_ (pH 1)	TiO_*x*_@MnO_*x*_	20	∼0.5@1.5	FDCA	25 (60°C)	NA	25 (60°C)	[81]
				∼0.8@1.6					
	0.05 M H_2_SO_4_ (pH 1)	δ‐MnO_2_	10	∼3@1.6	FDCA	NA	NA	46.8 (45°C)	[82]
				∼5@1.7	MA	NA	NA	42 (45°C)	[82]
	0.5 M H_2_SO_4_	Ag/AgO_*x*_–CN_*x*_	10	10@1.453	FDCA	72.4	NA	NA	[83]
	0.1 M Na_2_SO_4_ (pH 2.4)	Ru^II^(dipic‐PO_3_H_2_) (bipy)_2_	10	∼0.4@1.5 (CV)	DFF	∼78	NA	∼66	[84]
		[Ru^III^(dipic‐PO_3_H_2_) (bipy)Cl]	10	∼1.6@1.5 (CV)	FDCA	∼85	NA	85	[84]

Abbreviations: DFF, 2,5‐diformylfuran; FDCA, 2,5‐furandicarboxylic acid; FE, Faradaic efficiency; FFCA, 5‐formyl‐2‐furancarboxylic acid; HMFCA, 5‐hydroxymethyl‐2‐furancarboxylic acid; LSV, linear sweep voltammetry; MA, maleic acid; MOF, metal‐organic framework.

The rapid advancement in electrocatalytic HMF oxidation is evidenced by a growing body of review literature. Previous reviews have made valuable contributions by comprehensively summarizing reaction pathways and mechanisms,[^2^, ^5^] with a particular focus on FDCA synthesis,[^88^, ^89^] cataloging the development of diverse electrocatalysts including alloys,^[90]^ and outlining broader roadmaps for electrochemical biomass valorization.[^4^, ^10^] However, these discussions often fragment the influence of electrolyte pH within specific contexts, lacking a unified and in‐depth analysis that places pH dependency as the central governing factor. A consolidated understanding of how pH universally dictates reaction mechanisms, catalyst stability, and ultimate application potential remains a critical gap.

Addressing this fundamental gap requires a mechanistic understanding of HMFOR that explicitly accounts for pH effects. Core steps of the oxidation reaction—including initial dehydrogenation, hydroxymethyl/aldehyde group oxidation, dehydration, and potential side reactions—are inherently linked to system pH.[^16^, ^22^, ^88^] The protonation states and reactivities of HMF and its key intermediates (HMFCA, DFF, FFCA); the formation and charge state of species on the catalyst surface (e.g., formation of M‐OH or M‐OOH); and the thermodynamics and kinetics of proton‐coupled electron transfer (PCET) steps are all profoundly governed by pH.[^17^, ^20^, ^26^, ^28^, ^38^, ^91^] The current lack of systematic elucidation of these pH‐dependent mechanisms hinders the rational design of efficient electrocatalysts. Therefore, this review is structured to first systematically delineate and contrast the dominant reaction mechanistic models for HMFOR in alkaline, near‐neutral, and acidic media, elucidating how pH dictates the primary reaction pathways, rate‐determining steps, and the fundamental reasons for selectivity disparities. Building upon this mechanistic foundation, we will provide a comprehensive overview of pH‐specific catalyst design strategies, spanning multiple dimensions such as single‐atom catalysts (SACs), elemental doping, defect engineering, interface engineering, crystal structure engineering, and microenvironment modulation. Additionally, the reactor design suitable for HMFOR will be systematically summarized and discussed. By establishing clear correlations between the pH‐dictated reaction environment and the corresponding catalyst structure‐performance requirements, this review aims to outline a roadmap for accelerating the development of efficient and stable electrocatalysts functional under targeted pH conditions, thereby unlocking the full potential of HMF as a renewable feedstock.

## 2 OVERVIEWS OF REACTION MECHANISMS

The reaction pathway and efficiency of HMFOR are highly dependent on the operating conditions, particularly the electrolyte pH, and the physicochemical properties of the electrocatalyst. The core reaction mechanisms can be categorized into the “indirect oxidation mechanism (IOM)” and the “direct oxidation mechanism (DOM)” based on the mode of electron transfer and the role of the catalyst during the reaction; these mechanisms may coexist to form a mixed mechanism. Furthermore, the reactivity of key intermediates (HMFCA, DFF, FFCA) and their subsequent reaction pathways are strongly influenced by the system pH. This section will systematically elucidate these primary oxidation mechanisms and the decisive influence of pH on pathway selectivity.

**FIGURE 2:**
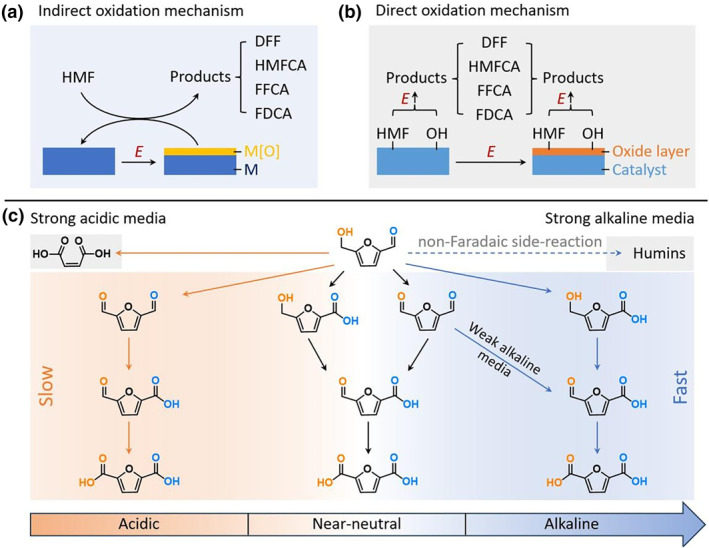
Schematic diagram of (a) the indirect oxidation mechanism and (b) the direct oxidation mechanism of HMFOR. (c) Schematic diagram of the common HMF conversion pathways in acidic, near‐neutral, and alkaline media. HMFOR, 5‐hydroxymethylfurfural oxidation reaction.

As illustrated in Figure 2a, the IOM involves applied potentials that do not directly oxidize HMF or organic intermediates. Instead, they preferentially drive the ECO of the catalyst itself (e.g., M → M[O]), generating highly oxidized metal species with strong oxidizing capabilities (e.g., NiOOH, CoOOH).[^16^, ^37^, ^50^, ^71^] These electrochemically “activated” high‐valence metal oxide species subsequently attack and convert adsorbed HMF or its organic intermediates through chemical oxidation (a non‐electrochemical step), becoming reduced themselves. The reduced catalyst species are then re‐oxidized by the applied potential, completing the catalytic cycle. The rate of this pathway may be limited by the formation rate of the high‐valence metal oxide species and their chemical oxidizing power. For instance, Zhou et al. reported that loading Pt onto Ni(OH)_2_ optimized the redox properties of Ni(OH)_2_, thereby facilitating the formation of the active NiOOH species to accelerate HMFOR.^[50]^ Similarly, Shi et al. found that doping Mn into amorphous NiFe alloy significantly lowered the onset potential for generating active Ni^3+^ species, which spontaneously reacted with HMF via nucleophilic dehydrogenation to form FDCA.^[37]^ Additionally, mediator molecules like 2,2,6,6‐tetramethylpiperidine‐1‐oxyl (TEMPO) are frequently employed to mediate HMF oxidation,[^66^, ^67^, ^68^] but this falls outside the primary focus of our discussion.

The DOM centers on the direct adsorption of HMF (or other intermediates) and oxidation species (such as *OH) onto active sites of the catalyst, where direct electron transfer across the electrode interface results in oxidation (Figure 2b). The catalyst's oxidation state remains largely unchanged throughout the HMF oxidation process. Its principal functions are to lower the activation energy barrier for specific functional groups, form and stabilize key reaction species (*HMF, *OH, etc.), and facilitate efficient electron transfer. For instance, in the Rh‐O_5_/Ni(Fe) structured catalyst developed by Zeng et al., single‐atom Rh sites selectively adsorb and activate HMF molecules while adjacent Ni sites generate electrophilic *OH species to effectuate oxidation, with strong d‐d orbital coupling between Rh and Ni significantly enhancing electron transfer capability to synergistically enable efficient HMF oxidation.^[32]^ Of course, the catalyst may sometimes undergo ECO first; the resulting oxidized species then act as electrocatalysts for the direct ECO of HMF. For instance, Wu et al. electrochemically reconstructed a 5‐nm thick (Ni, Mo)OOH layer on NiMo_3_S_4_.^[29]^ This in situ formed ultrathin (Ni, Mo)OOH layer with sulfate‐terminated species enhanced the adsorption of HMF and OH^−^ as well as charge transfer, consequently boosting HMFOR activity.

In most practical HMFOR systems, direct and IOMs are often likely to coexist. Different reaction steps (e.g., oxidation of distinct functional groups or intermediates) may follow different mechanisms. For instance, the initial oxidation of the aldehyde group might occur via DOM, while the further oxidation of certain intermediates (e.g., HMFCA) or the oxidation of the hydroxymethyl group might rely more heavily on chemical oxidation by surface‐generated high‐valence oxidized species (IOM).^[5]^

Electrolyte pH profoundly influences the protonation state of reactants/intermediates, catalyst surface species, and adsorption modes, thereby governing the dominant reaction pathway and product selectivity. Depending on the pH of the electrolyte, the HMF oxidation pathway differs, hinging critically on whether the hydroxymethyl group or the aldehyde group of HMF undergoes oxidation first.[^2^, ^5^, ^90^] In strong alkaline media (pH ≥ 13), the aldehyde group of HMF is preferentially oxidized, and its conversion to FDCA typically follows the HMF‐HMFCA‐FFCA‐FDCA pathway (Figure 2c). Specifically, the aldehyde group rapidly undergoes hydration in the presence of high OH^−^ concentration to form the more reactive geminal diol anion, which readily undergoes efficient dehydrogenation, via chemical oxidation by high‐valence surface oxidizing species or direct electron transfer, to the carboxylic acid (HMFCA).[^64^, ^92^] As the electrolyte pH decreases, oxidation of the hydroxymethyl group becomes increasingly prominent. In our previous work, it was found that in a weak alkaline system (pH 11), both the aldehyde and hydroxymethyl groups of HMF could be oxidized during the initial stages.^[16]^ Consequently, besides the HMF‐HMFCA‐FFCA‐FDCA path, the HMF‐DFF‐FFCA‐FDCA path also existed, and the latter was found to be dominant.^[16]^

For near‐neutral systems (pH 5–9), the HMFOR mechanism is more complex than in alkaline systems. The lack of sufficient OH^−^ to promote aldehyde activation in near‐neutral media narrows the energy barrier difference between hydroxymethyl and aldehyde oxidation. This allows both the aldehyde pathway (HMF → HMFCA) and the hydroxymethyl pathway (HMF → DFF) to potentially occur and compete initially (Figure 2c). Subsequent oxidation steps (HMFCA → FFCA → FDCA or DFF → FFCA → FDCA) are also suppressed. Under these conditions, product selectivity is typically dictated collectively by the electrocatalyst material, electrolyte composition, applied potential, reaction time (charge passed), etc. For example, by controlling these reaction conditions, HMFCA, DFF, FFCA, or FDCA can each be obtained as the main product in near‐neutral systems.[^17^, ^69^, ^70^, ^79^]

As the electrolyte pH decreases further into the acidic range (pH ≤ 5), hydroxymethyl oxidation generally dominates, and HMF conversion to FDCA follows the HMF‐DFF‐FFCA‐FDCA pathway (Figure 2c).[^6^, ^80^] However, it is important to note that oxidation of the aldehyde groups in DFF/FFCA proceeds very slowly with poor selectivity under acidic conditions, often resulting in only partial oxidation to FFCA/FDCA or even stagnation, making high FDCA selectivity and yield difficult to achieve.[^6^, ^80^, ^81^] This can be attributed partly to the inherently high activation barrier for aldehyde oxidation in acidic media and the lack of efficient activation pathways, and partly to the extreme difficulty in forming and maintaining stable, highly active high‐valence metal oxide species (M[O]) in acidic environments, which severely compromises oxidizing capability. Furthermore, under applied potential and strongly acidic conditions (e.g., pH ≤ 1), the furan ring of HMF becomes susceptible to an electrochemically driven acid hydrolysis, leading to the formation of MA as a by‐product.[^6^, ^82^] This ring‐opening pathway competes with the electrooxidation reaction, significantly compromising FDCA yield under such conditions.

In summary, this section systematically discusses the dominant electrocatalytic pathways of HMFOR under different pH conditions and elucidates their underlying mechanisms. Understanding these distinctions is fundamental toward developing efficient HMFOR electrocatalysts. The following section will summarize and discuss cutting‐edge catalyst design strategies that specifically address these pH‐specific challenges.

## 3 pH‐DEPENDENT CATALYST DESIGN STRATEGIES

### 3.1 Alkaline media

As discussed previously, HMFOR kinetics are rapid in alkaline media with the main product predominantly being FDCA, only a handful of reports yield HMFCA as the major product. In this section, we will summarize and discuss advanced HMFOR electrocatalyst design strategies for alkaline systems, including SACs, elemental doping, defect engineering, interface engineering, crystal structure engineering, and microenvironment modulation. Additionally, we will briefly discuss reactor design to meet practical application requirements.

#### 3.1.1 Single‐atom catalysts

Designing efficient SACs for HMFOR in alkaline media hinges on addressing the issue of competitive adsorption between reactant molecules (HMF and its oxidation intermediates) and the abundant OH^−^ ions present in the electrolyte at the catalyst's active sites. Literature widely reveals that the introduction of single atoms offers a unique pathway for finely tuning the electronic structure and adsorption properties of the support material.[^31^, ^32^, ^33^, ^93^, ^94^] For instance, whether Lu et al. introduced Ir onto Co_3_O_4_ ^[31]^ or our group introduced Ru,^[33]^ the core mechanism involves optimizing the support's adsorption behavior towards key reactive species (HMF molecules and OH^−^ ions) via the single‐atom sites. Single atoms can not only serve as additional adsorption sites but also significantly alter the local charge density and electronic states of the support, thereby tailoring its affinity and adsorption energy for different reactants. This atomic‐level tuning enables the catalyst to preferentially or cooperatively adsorb the required species in complex reaction environments. Examples include enhancing key OH^−^ adsorption under low OH^−^ concentrations (as in our group's work^[33]^) to maintain reaction kinetics or promoting critical reaction steps (such as DFF formation, as in Xu et al.'s work^[94]^) while retaining high HMF adsorption capacity. This fundamentally mitigates the limitation imposed by competitive adsorption on reaction efficiency.

Current SAC design strategies are evolving beyond solely optimizing HMFOR performance towards integrating multiple functionalities (e.g., coupling the hydrogen evolution reaction [HER]). The atomically dispersed co‐adsorption centers designed by Zeng et al.^[32]^ (where Rh adsorbs and activates HMF, and Ni provides *OH) and the exploration of bifunctional catalysts (simultaneously efficient for HMFOR and HER) by Cui et al.^[93]^ collectively highlight the advanced strategy of constructing spatially or electronically separate (or cooperative) multi‐active sites within a single catalyst system. This facilitates the efficient cascade of complex reaction pathways and the coupling of multiple functionalities. This design effectively improves reaction efficiency, exemplified by Zeng et al. achieving hydrogen co‐production at a low cell voltage of 1.48 V@100 mA cm^−2^.^[32]^ Collectively, these studies indicate that ideal HMFOR SAC design requires synergistic coordination between single‐atom sites and the support to precisely balance adsorption energies for key reactive species, construct efficient atomically dispersed bifunctional centers, and simultaneously ensure catalytic stability, thereby paving the way for biomass electrocatalytic refining industrialization.

#### 3.1.2 Elemental doping

**FIGURE 3:**
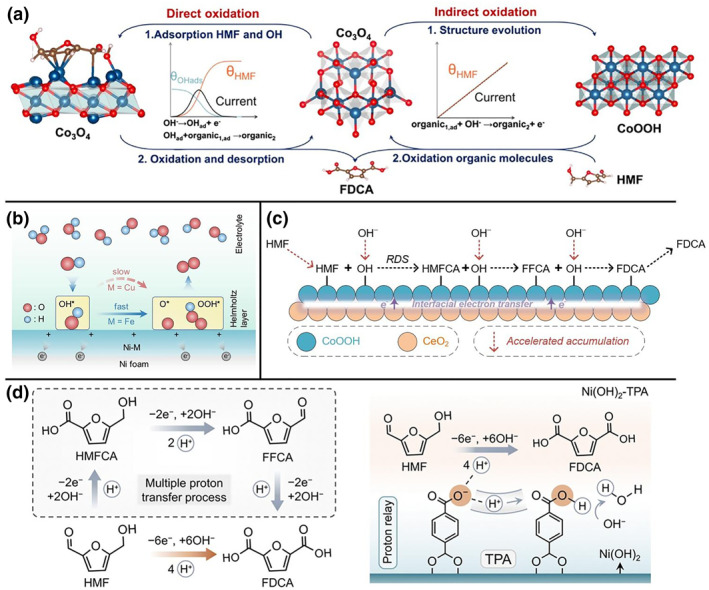
(a) Schematic representation of the direct and indirect oxidation of HMF.^[34]^ (b) Schematic illustration of intermediates evolution over Ni−M/NF during OER.^[57]^ (c) Schematic diagram of the HMFOR process on CoOOH@CeO_2_.^[26]^ (d) Reaction route of HMF oxidation to FDCA under strong alkaline conditions (left) and bio‐inspired proton relay through the TPA ligand of Ni(OH)_2_‐TPA (right).^[60]^ FDCA, 2,5‐furandicarboxylic acid; HMFOR, 5‐hydroxymethylfurfural oxidation reaction; OER, oxygen evolution reaction; TPA, terephthalic acid.

In alkaline HMFOR, the core efficacy of elemental doping lies in the precise regulation of the catalyst's electronic structure. This optimization fine‐tunes the adsorption energy of the active sites towards the key functional groups of HMF (hydroxymethyl and aldehyde) and OH^−^ ions, thereby efficiently driving either the direct or indirect oxidation pathways (Figure 3a). For instance, Lu et al. revealed that Co_3_O_4_ exhibits high activity towards the aldehyde group while NiO shows strong affinity for the hydroxymethyl group.^[34]^ Therefore, incorporating Ni into the tetrahedral sites of Co_3_O_4_ to form Ni_0.5_Co_2.5_O_4_ optimized the adsorption matching for both functional groups, significantly enhancing the efficiency of the direct oxidation pathway (FDCA yield >92%). In addition, the indirect oxidation pathway relies on electrochemically generated high‐valent metal oxide species (e.g., Ni^3+^‐O or Co^3+^‐O) acting as chemical oxidants to attack the HMF molecule. For example, Wu et al. found that Pt doping into Ni not only accelerated the ECO of Ni^2+^ to the crucial Ni^3+^ active species (the initiation step of the indirect pathway) but also, through the formation of Pt‐O‐Ni bonds, promoted OH^−^ adsorption on Ni sites (providing the oxygen source for subsequent dehydrogenation).^[51]^ Similarly, Shi et al. demonstrated that Mn doping in amorphous NiFeB enhanced oxygen intermediate adsorption and significantly lowered the onset potential for Ni^3+^ formation, substantially boosting the selectivity and kinetics of the indirect oxidation pathway while effectively suppressing the oxygen evolution reaction (OER) competition.^[37]^

Addressing industrial requirements, elemental doping strategies hold potential to tackle the core challenges of achieving high current density, high selectivity, and system energy efficiency. For example, Mn‐doped NiS prepared by Li et al. achieved industrial‐scale current density (500 mA cm^−2^) while delivering high FDCA yield (97.6%), selectivity (98.3%), and space‐time yield (44.32 g h^−1^ in a flow cell).^[36]^ Co‐doped Ni_3_S_2_/NF reported by Sun et al. also demonstrated a high current density reaching 497 mA cm^−2^ ^[35]^ More significantly, in coupled hydrogen production systems, optimized doped catalysts (e.g., NiCoB_*x*_ reported by Xu et al.^[30]^), by efficiently driving HMFOR to replace the OER, can enable simultaneous high‐yield FDCA and H_2_ production at low cell voltages (1.62 V@400 mA cm^−2^), substantially reducing the energy consumption for hydrogen production (saving ∼1.03 kWh/m^3^ H_2_). In summary, elemental doping provides a crucial solution pathway for designing efficient and stable alkaline HMFOR electrocatalysts tailored to different reaction routes (direct HMF activation or mediated indirect oxidation) achieved through the targeted modulation of the adsorption properties and redox behavior of the reaction sites.

#### 3.1.3 Defect engineering

Defect engineering serves as a pivotal strategy for enhancing the performance of HMFOR electrocatalysts. The deliberate introduction of various defects, including anionic defects (such as oxygen vacancies), cationic defects, and lattice defects, can significantly optimize the catalyst's electronic structure, augment the electrochemically active surface area, expose a greater number of unsaturated active sites, and effectively modulate the adsorption behavior of key reactants (HMF and OH^−^).[^39^, ^40^, ^41^, ^42^, ^43^, ^54^] These synergistic modifications collectively promote efficient charge transfer, lower the reaction energy barrier, and thereby substantially elevate catalytic activity and FE while enhancing reaction stability. For instance, Song et al. demonstrated that constructing abundant oxygen vacancies within ultrathin CoAl‐LDH (layered double hydroxide) layers effectively reduced the onset potential to 1.30 V_RHE_ and achieved FDCA FE exceeding 95% over a broad potential window.^[40]^

It is noteworthy, however, that defect introduction necessitates precise modulation. An excessively high defect concentration can induce the over‐adsorption of reactants (HMF or OH^−^), potentially inhibiting the catalytic process.^[44]^ To address this, researchers have developed innovative “defect regulation” strategies. One approach involves modifying defect sites with noble metals or heteroatoms to tune the local electronic environment. For example, Zhan et al. loaded Pt onto Co_3_O_4_ metal defect sites (forming Pt‐V_CO_), inducing an upward shift in the d‐band center and forming Pt‐Co bonds.^[45]^ This synergistic effect enhanced the adsorption and activation capabilities for both HMF and OH^−^, resulting in an exceptionally low onset potential of 1.14 V_RHE_ and a 19‐fold increase in current density at 1.35 V_RHE_. Additionally, a complementary “defect filling” strategy was employed. Cheng et al. introduced S atoms into LDHs enriched with oxygen vacancies, creating a S‐O_V_‐LDH structure.^[44]^ This effectively attenuated the overly strong adsorption induced by excess oxygen vacancies, optimized the adsorption strength of reaction intermediates, and promoted the formation of highly active Co^3+^ species. These strategies underscore the paramount importance of precisely modulating defect type, concentration, and local electronic structure as the core principle for realizing high‐performance HMFOR electrocatalysts.

#### 3.1.4 Interface engineering

Interface engineering stands as a sophisticated catalyst design strategy that significantly enhances the HMFOR performance by constructing heterointerfaces to effectively modulate the electronic structure and surface properties of catalysts. For instance, the CoOOH@CeO_2_ heterostructure reported by us (formed via in situ reconstruction of CoBDC@CeO_2_) exhibits exceptional catalytic activity.^[26]^ The introduction of the CeO_2_ component optimized the interfacial electronic structure, facilitated electron transfer, accelerated the enrichment of the key intermediate *OH, and markedly reduced the reaction energy barrier (Figure 3c). Consequently, the potential required for HMFOR was substantially reduced compared to that for the OER (△*E* = 222 mV@50 mA cm^−2^), while simultaneously achieving high FDCA selectivity and FE.

The fundamental significance of heterointerfaces lies in their precise regulation of the adsorption behavior of key reactive species and a notable improvement in electron transfer efficiency. Combined theoretical calculations and experimental characterizations reveal that, for example, CeO_2_ functions as an “electron pump” within the Co_4_N system, drawing electron flow towards the CeO_2_ side and optimizing the adsorption energy for both OH^−^ and HMF.^[18]^ Similarly, heterointerfaces such as CuO‐PdO,^[19]^ NiS_*x*_/Ni(OH)O,^[20]^ NiCo_2_@MoO_2_,^[21]^ and Co_3_O_4_/CeO_2_ ^[22]^ enhance the catalyst's adsorption and activation capabilities towards HMF molecules and OH^−^ species through robust interfacial electronic interactions. This optimized adsorption not only accelerates reaction steps, including the PCET process but also lowers the activation energy of critical steps like FFCA dehydrogenation, thereby improving HMFOR kinetics and overall performance.

Interface engineering further profoundly influences the dynamic structural evolution of catalysts and their reaction pathways. Heterostructures may undergo structural reconstruction under electrocatalytic conditions or activate high‐valent metal active species (e.g., Ni^2+δ^ species).[^28^, ^50^] Taking Pt‐modified Ni(OH)_2_ as an example, the introduction of Pt not only optimized the redox properties of Ni(OH)_2_, accelerating the formation of Ni(OH)O (the true HMFOR active species derived from its dehydration), but the Pt sites themselves also served as preferential adsorption sites for HMF molecules, optimizing the adsorption behavior.^[50]^ Concurrently, such interface‐mediated dynamic processes (e.g., the NiOOH‐mediated dual‐cycle mechanism revealed in the Ni(OH)_2_‐NiOOH/NiFeP heterojunction^[28]^) are crucial for understanding and regulating the competitive relationship between HMFOR and the OER, guiding the reaction towards efficient progression.

Furthermore, meticulously designed heterointerfaces can confer additional functional advantages and enable selective product regulation. The Ag‐Co(OH)_2_ demonstrated the capability to selectively synthesize either HMFCA or FDCA—both high‐value‐added products—within a single catalyst system by applying different potential ranges.^[95]^ The incorporation of Ag activated the cobalt‐based compound, strengthening adsorption of organic molecules and OH^−^ species. This promoted oxidation of the aldehyde group to a carboxyl group at lower potentials (yielding HMFCA) while favoring FDCA formation at higher potentials. More remarkably, heterointerfaces featuring optimized electronic structures (e.g., Co_4_N@CeO_2_) can even enable efficient multifunctional catalysis (HER/OER/HMFOR trifunctional activity), presenting opportunities for coupled systems, such as HMFOR‐assisted H_2_ production.^[18]^ This not only enhances the economic value of HMFOR but also offers a valuable strategy towards achieving carbon neutrality goals. Collectively, these studies systematically elucidate the mechanisms through which heterointerfaces strengthen adsorption, regulate electronic structure, and optimize reaction pathways, providing clear guidance for designing next‐generation high‐performance HMFOR electrocatalysts.

#### 3.1.5 Crystal structure engineering

The essence of crystal structure engineering lies in the precise modulation of the spatial arrangement and electronic properties of active sites. Song et al. revealed the directional influence of crystal facet exposure on the reaction pathway.^[48]^ Specifically, the NiO(111) facet enhances HMF adsorption through d‐π conjugation arising from continuous low‐valent Ni sites, while its unique atomic arrangement significantly lowers the adsorption energy barrier for hydroxyl species, thereby addressing the kinetic coupling challenge of substrate adsorption and hydroxyl supply in HMFOR. Similarly, Zhang et al. discovered that modulating the exposed crystal facets of Co_3_O_4_ could alter its adsorption and catalytic capabilities toward HMF.^[96]^ Lu et al. demonstrated that Co^3+^ octahedral sites play a crucial role in HMFOR, and the introduction of Cu^2+^ can selectively expose these Co^3+^ sites and strengthen organic molecule adsorption, overcoming the traditional bottleneck of bifunctional site isolation in cobalt‐based catalysts.^[46]^ Moreover, Zhou et al. uncovered the characteristic of lattice‐strain‐induced dynamic generation of NiOOH within NiCo_2_O_4_, revealing that the crystal structure is not merely a static template.^[47]^ Instead, the electric field can synergistically cooperate with lattice distortion to in situ construct active species, providing a novel perspective on catalyst dynamic reconstruction.

Moving beyond single facet modulation, structural engineering in multicomponent materials emerges as a key strategy for enhancing FDCA production efficiency. Zhu et al. reported that a sub‐nanoscale Cu‐Co_3_O_4_ gradient structure establishes an internal built‐in electric field by inducing lattice distortion, enabling charge transfer efficiency to surpass the limitations imposed by graphene encapsulation layers.^[49]^ This principle of multiscale design is epitomized within metal‐organic framework systems. For instance, the PW_12_@Ni‐HITP composite reported by Bao et al. constructs bifunctional active centers bridged by Ni‐O‐W bonds, with its ordered channels achieving directional diffusion and separation of HMF oxidation products.^[55]^ Similarly, the glassy ZIF‐CoNi material reported by Shen et al. leverages weakened Co‐N bonds to trigger in situ electrochemical reconstruction, generating an active CoOOH layer.^[27]^ Collectively, these advancements demonstrate that an ideal HMFOR catalyst necessitates the integration of tripartite design features: (1) atomic‐scale active site engineering (facet/site control), (2) mesoscale mass transport channels (arrays/porosity), and (3) cross‐interface charge transfer networks (gradient components/bonded structures). Future breakthroughs will depend on developing crystal structures that simultaneously possess dynamic response capabilities and deliver industrial‐level current densities.

#### 3.1.6 Microenvironment modulation

Suppressing the competing OER for active sites and electrons represents a key challenge for enhancing HMFOR efficiency in alkaline media.[^57^, ^58^, ^61^] Microenvironment regulation offers a fundamental solution, focusing on the precise modulation of adsorption behaviors and reaction energy barriers at the electrode surface. For instance, Chen et al. reported that by constructing a Ni‐Cu catalyst, they successfully modulated the reaction microenvironment at the electrode‐electrolyte interface, effectively “passivating” the OER while promoting the HMFOR (Figure 3b).^[57]^ Wang et al. proposed an adsorption microenvironment remodeling strategy, utilizing carbonate species (adsorbed ${\text{CO}}_{3}^{2-}$ derived from the in situ decomposition of nickel oxalate) to modify the NiOOH surface.^[61]^ These adsorbed ${\text{CO}}_{3}^{2-}$ anions significantly reduce the interfacial OH^−^ coverage through electrostatic repulsion, physically freeing up adsorption space for HMF molecules, while theoretical calculations also indicate they increase the energy barrier for the *O to *OOH transformation. Collectively, these studies elucidate that effectively suppressing OER may necessitate fundamentally altering the intrinsic reactivity of catalytic sites towards key oxygen intermediates.

Achieving high‐current‐density, efficient HMFOR with maintained system stability—without compromising selectivity—demands the synergistic optimization of the reaction interface to facilitate mass transport and charge transfer.[^59^, ^60^] The ligand‐modified catalyst (Ni(OH)_2_‐TPA) designed by Chen et al. exemplifies this approach.^[60]^ Non‐coordinating carboxyl functional groups on the TPA ligands construct an efficient proton transport network near the active sites, functioning as proton‐relay centers (Figure 3d). This significantly accelerates the PCET kinetics during oxidation, enabling the maintenance of high FE even at industrial‐level current densities (1000 mA cm^−2^). Additionally, the work by Wu et al. directly addresses the challenge of catalyst structural instability under high‐current operation.^[29]^ Their “self‐confined surface reconstruction” strategy by pre‐incorporating stabilizing elements (Mo, S) within the NiMo_3_S_4_ substrate successfully limits the thickness of the reconstructed NiOOH layer to approximately 5 nm (∼5 nm). This drastically enhances electron conduction rates and prevents deactivation caused by deep oxidation. These advancements indicate that suppressing the OER, integrating proton transfer promotion, optimizing the electronic structure, and employing nanostructure confinement are core directions for constructing advanced HMFOR electrocatalytic microenvironments exhibiting both high activity and exceptional stability.

#### 3.1.7 Emerging focus in alkaline systems

**FIGURE 4:**
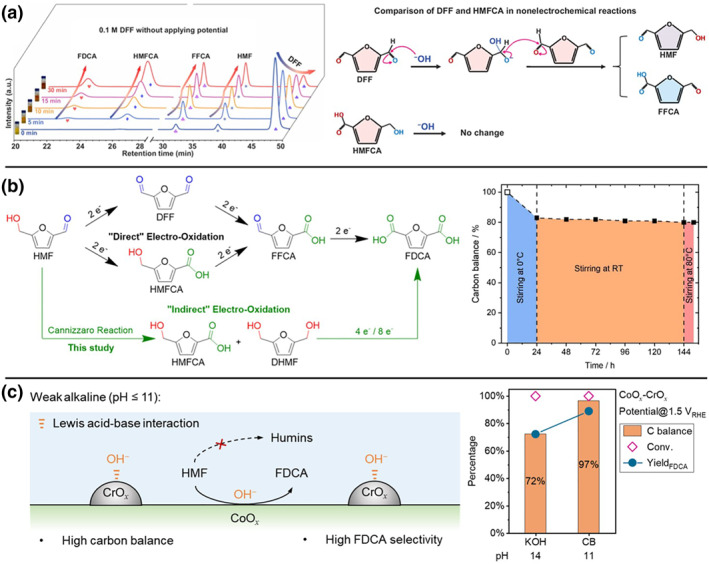
(a) HPLC chromatograms for the nonelectrochemical reaction of DFF obtained without applying potential over A‐Co‐Ni_2_P catalyst, and comparison of DFF and HMFCA in nonelectrochemical reactions under alkaline conditions.^[65]^ (b) Reaction scheme of electrochemical HMF oxidation in alkaline media, and carbon balance of a 1 M HMF solution in 5 M KOH.^[97]^ (c) HMF electrooxidation in weak alkaline media (pH ≤ 11) catalyzed by CoO_*x*_‐CrO_*x*_, and the carbon balance, HMF conversion and FDCA yield in KOH and CB media.^[16]^ CB, carbonate buffer; DFF, 2,5‐diformylfuran; FDCA, 2,5‐furandicarboxylic acid; HMFCA, 5‐hydroxymethyl‐2‐furancarboxylic acid; HPLC, high‐performance liquid chromatography.

A core challenge facing alkaline HMFOR systems is the degradation of HMF and its key intermediates induced by the high‐pH environment, such as the formation of intractable humins. Spatially separated experiments combined with high‐performance liquid chromatography monitoring by Chen et al. confirmed that the intermediate DFF readily undergoes spontaneous Cannizzaro disproportionation in strong alkali, rapidly generating equimolar amounts of HMF and FFCA, which are subsequently converted to HMFCA and FDCA.^[65]^ This process, accompanied by a visible color change of the solution from yellow to red, starkly highlights the instability of DFF, whereas HMFCA remained stable under identical conditions (Figure 4a).

To overcome HMF alkaline degradation and enhance FDCA synthesis efficiency, researchers have explored innovative countermeasures. Krebs et al. observed that alkaline degradation products HMFCA and dihydroxymethylfuran (DHMF) themselves exhibit good stability in strong alkali, and both can be effectively electrooxidized to the final product FDCA (Figure 4b).^[97]^ This finding inspired a novel oxidation strategy: even under high substrate and high alkali concentrations (significantly exceeding conventional operating conditions) stable intermediates generated from HMF degradation (HMFCA and DHMF) are prioritized for subsequent electrochemical conversion. This approach successfully circumvented the severe degradation encountered during the direct electrooxidation of HMF, achieving near‐practical current densities (∼1 A cm^−2^), thereby greatly advancing the technical feasibility of this process. Furthermore, our group focused on boosting catalytic activity within a weak alkaline medium, which effectively suppressed HMF degradation (Figure 4c).^[16]^ We addressed the activity bottleneck arising from low OH^−^ concentration under weak alkaline conditions (pH 11, carbonate buffer) by constructing a CoO_*x*_‐CrO_*x*_ catalyst through the introduction of hard Lewis acid oxide (CrO_*x*_) onto the CoO_*x*_ surface. The incorporation of CrO_*x*_ was demonstrated to significantly accelerate the migration of OH^−^ from the bulk electrolyte to the electrode surface and concentrate OH^−^ on the electrode‐electrolyte interface. This not only dynamically optimized the interfacial microenvironment and facilitated catalyst reconstruction but also enhanced the conversion rate of intermediates. Consequently, under weak alkaline conditions (with carbon balance up to 97%), we achieved high FDCA FE (90%), selectivity (92%), and yield (89%) while tolerating HMF initial concentrations as high as 100 mM. Additionally, studies have reported that 2,5‐bis(hydroxymethyl)furan (BHMF) can be used as an alternative to HMF for the synthesis of FDCA, thereby avoiding non‐Faradaic side reactions of HMF.[^98^, ^99^]

These studies reveal emerging principles and focus areas for HMFOR catalyst design in alkaline media. On one hand, a deepened understanding and utilization of the inherent chemical stability of substrates/intermediates in alkali (e.g., HMFCA stability) or even their degradation pathways (e.g., disproportionation products), can lead to the development of novel conversion pathways operating effectively at high concentrations and currents.^[97]^ On the other hand, tackling the fundamental conflict where weak alkali suppresses HMF degradation but limits activity, necessitates the design of sophisticated catalyst surface structures (e.g., introducing hard Lewis acid species) to locally dynamically enrich OH^−^, thereby efficiently activating the electrocatalyst surface and accelerating intermediate conversion.^[16]^ This provides a promising new direction for maintaining electrocatalytic activity while suppressing carbon loss. Additionally, Fu et al. investigated the unique geminal diol intermediates of HMF in alkaline media (DHMFM^−^ and DHMFM^2−^) and their oxidation pathways (producing either H_2_ or H_2_O).^[64]^ This work deepens the understanding of the reaction's microscopic mechanism and offers a potential theoretical basis for regulating the selectivity of the oxidation process.

### 3.2 Near‐neutral media

Compared to alkaline conditions, the HMFOR in near‐neutral media (pH 5–9) presents unique challenges and opportunities. This pH range avoids extreme corrosiveness while enhancing the stability of HMF and its intermediates, offering improved environmental compatibility. However, it also significantly increases the complexity of catalytic pathways. Here, the limited OH^−^ concentration makes the reactivity, solvation, and adsorption configurations of HMF and its oxidation intermediates on catalyst surfaces highly sensitive and delicate. This directly leads to intricate reaction pathways and diverse product distributions. Consequently, precise control over product selectivity—particularly the stable generation of specific target products (such as DFF, HMFCA, FFCA, or FDCA)—emerges as both the central objective and key challenge in designing near‐neutral HMFOR catalysts. Given the distinct synthesis pathways and catalyst requirements for different target products, this section will systematically explore critical strategies and regulatory approaches for achieving high selectivity in near‐neutral media, using the main electrooxidation products as the classification criterion.

#### 3.2.1 DFF as the main product

**FIGURE 5:**
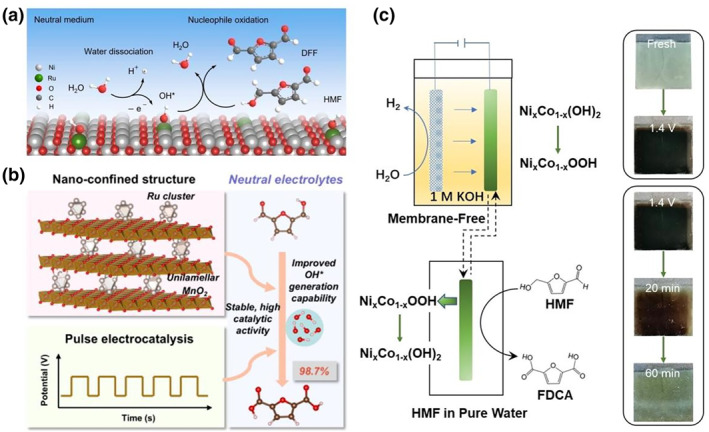
(a) Proposed HMFOR mechanism over Ru_1_‐NiO in the neutral medium.^[17]^ (b) The sandwich catalyst composed of Ru clusters and monolayer MnO_2_ achieves FDCA production in neutral media through a combination of nano confinement and electrochemical pulses.^[69]^ (c) Decoupling system schematic diagram, in which Ni_*x*_Co_1‐*x*_(OH)_2_ is used as a redox mediator to achieve FDCA production in pure water, and digital photographs of the Ni(OH)_2_/FTO surface color change during the decoupling system.^[71]^ FDCA, 2,5‐furandicarboxylic acid; HMFOR, 5‐hydroxymethylfurfural oxidation reaction.

Electrocatalytic oxidation of HMF to selectively produce the high‐value product DFF in near‐neutral media represents a critical research direction in the HMFOR field. The primary challenge lies in suppressing the over‐oxidation of the aldehyde group to prevent its further conversion into carboxylic acid derivatives. Notably, near‐neutral conditions inherently favor the stability of aldehyde groups compared to strongly alkaline environments, providing a fundamental advantage in addressing this challenge. A breakthrough strategy was demonstrated by our group,^[17]^ we designed a nickel oxide‐supported single‐atom ruthenium (Ru_1_‐NiO) catalyst that exhibited exceptional performance under near‐neutral conditions. This catalyst required a low working potential of 1.283 V_RHE_ at a current density of 10 mA cm^−2^ while achieving a remarkable DFF selectivity of up to 90%. Mechanistic studies revealed that the single‐atom Ru sites efficiently promoted water dissociation to generate *OH active species, significantly enhancing the HMF oxidation rate in neutral media. Concurrently, the inherent properties of the near‐neutral environment effectively stabilized the formed aldehyde groups. The synergistic effect of these two factors ultimately enabled high DFF selectivity (Figure 5a). Furthermore, this catalytic system demonstrated excellent versatility, extending its applicability to the highly selective conversion of various biomass‐derived alcohols into their corresponding aldehyde derivatives.

In addition to Ru‐based SACs, other materials have demonstrated excellent DFF selectivity in near‐neutral media. For example, Le et al. developed an amorphous cobalt‐cerium composite oxide (Co_8_Ce_2_O_*x*_) catalyst that effectively suppressed aldehyde overoxidation under near‐neutral conditions, achieving high DFF selectivity ranging from 60% to 92%.^[77]^ Their study revealed that the incorporation of cerium optimized the electronic structure of the catalyst, facilitating the formation of active CoOOH/CoO_2_ species, while the near‐neutral environment played a crucial role in stabilizing the aldehyde group. Similarly, Zhou et al. reported a polyoxometalate‐based Ru_4_/PEI‐rGO composite catalyst, which achieved 66.2% DFF selectivity during HMF electrooxidation coupled with cathodic hydrogen evolution in near‐neutral conditions.^[78]^ These studies, employing different material systems, consistently highlight that the near‐neutral electrolyte environment serves as a fundamental factor in stabilizing primary HMF oxidation intermediates and enabling highly selective DFF synthesis.

#### 3.2.2 HMFCA as the main product

Achieving highly selective conversion of HMF to HMFCA in near‐neutral media requires precise regulation of active species and reaction potential to facilitate selective aldehyde group oxidation while effectively suppressing competitive hydroxymethyl oxidation. Xie et al. designed and synthesized a Co(OH)_2_‐CeO_2_ composite catalyst, demonstrating that under neutral conditions (pH 7) at 1.4 V_RHE_, this system efficiently catalyzes the specific two‐electron oxidation of HMF, converting the aldehyde group into a carboxyl group to produce HMFCA with remarkable selectivity (up to 89.4%).^[79]^ Their study revealed that the neutral pH environment and optimized anodic potential (1.4 V_RHE_) effectively inhibited the oxidation of the hydroxymethyl group on HMF while preventing further overoxidation to FFCA and FDCA. Moreover, this system successfully promoted the rapid formation and stabilization of critical Co^3+^ active species (e.g., CoOOH), which are essential for selectively catalyzing the two‐electron oxidation pathway of the HMF aldehyde group. Only when the potential increased sufficiently to generate more oxidizing Co^4+^ species (e.g., CoO_2_) did hydroxymethyl oxidation occur. Therefore, by precisely controlling reaction conditions (pH ∼7, potential ∼1.4 V_RHE_) to modulate the electronic structure and redox properties of catalytic active centers—thereby stabilizing key Co^3+^ active species—this strategy enables preferential aldehyde oxidation while suppressing hydroxymethyl oxidation, significantly enhancing HMFCA selectivity.

#### 3.2.3 FFCA as the main product

Selective oxidation of HMF to FFCA in near‐neutral media presents a critical challenge due to sluggish aldehyde oxidation kinetics caused by low OH^−^ concentration and insufficient catalyst adsorption capacity for HMF and its oxidation intermediates, which often leads to difficulty in FFCA accumulation. Lu et al. addressed this issue through an innovative strategy by designing a V_O_‐Co_3_O_4_ electrode with oxygen vacancies (V_O_).^[73]^ Their study revealed that in neutral phosphate buffer electrolyte (pH ∼7), HMF oxidation primarily proceeds via the DFF intermediate pathway. Compared to conventional Co_3_O_4_ electrodes, V_O_‐Co_3_O_4_ significantly accelerates the conversion of DFF to FFCA. This enhancement stems from the V_O_ on the electrode surface, which enable more efficient capture of scarce OH^−^ ions from the solution and promote their coupling with organic reaction intermediates. This mechanism highlights the importance of optimizing the local microenvironment of the electrode surface to enhance OH^−^ supply and activation, representing a key factor in improving FFCA selectivity in neutral media.

The weak adsorption of HMF and low oxidation activity of aldehyde groups in neutral media have been addressed through precise modulation of catalyst active sites via electronic structure engineering strategies. In the NiO‐PtO_*x*_ system reported by Wang et al.,^[76]^ the introduction of PtO_*x*_ induced electron redistribution and electronic structure optimization at Ni active sites, significantly enhancing HMF adsorption capability and achieving a high FFCA yield of 77% in neutral media. Similarly, Lu et al. developed a single‐atom Rh‐doped V‐NiO‐Rh/CF catalyst with Rh occupying Ni vacancies, where the single‐atom Rh reduced the electron density of Ni active sites.^[74]^ This not only optimized the HMF adsorption energy but also facilitated *OH species release via water dissociation by modulating the *OH adsorption energy barrier. Furthermore, studies by Choi et al.^[72]^ and Tao et al.^[75]^ confirmed that under near‐neutral conditions, HMF electrooxidation preferentially oxidizes the hydroxymethyl group to form DFF (due to faster alcohol oxidation kinetics), while subsequent aldehyde group oxidation of DFF to FFCA proceeds more slowly. This results in FFCA being the most readily accumulated product along this pathway, explaining the high FFCA selectivity observed in the aforementioned catalytic systems.

#### 3.2.4 FDCA as the main product

The primary challenge in achieving efficient HMF electrooxidation to FDCA in near‐neutral media lies in the sluggish reaction kinetics and low FDCA yield due to insufficient OH^−^ ions in the electrolyte. He et al. addressed this issue through an innovative synergistic strategy combining catalyst design and electrolysis techniques.^[69]^ They developed a sandwich‐structured catalyst (S‐Ru/MnO_2_), where Ru clusters confined between monolayer MnO_2_ nanosheets were coupled with pulsed electrolysis, significantly enhancing the performance in neutral systems (Figure 5b). The pulsed electrolysis dynamically maintained the low oxidation state of Ru active sites by modulating the applied potential, while strong electron transfer between Ru clusters and MnO_2_ nanosheets accelerated the generation of *OH active species. This approach achieved a breakthrough current density of 47 mA cm^−2^ and an exceptional FDCA yield of 98.7% at 1.55 V_RHE_. This work demonstrates that optimizing both catalyst design and electrolysis mode can simultaneously achieve high activity and high FDCA selectivity in neutral media.

To avoid the disproportionation and humification side reactions of HMF in strongly alkaline environments, the development of non‐alkali‐dependent electrolysis systems has become a crucial research direction. Zhang et al. proposed a “two‐step decoupling” strategy employing nickel‐cobalt hydroxide (Ni_0.85_Co_0.15_OOH) as a redox mediator, achieving efficient HMF conversion in pure water (Figure 5c).^[71]^ In this system, the metal hydroxide is first electrochemically oxidized to form high‐valent active species at the electrode surface, which subsequently selectively oxidize HMF molecules in aqueous solution, attaining nearly 100% FDCA selectivity.

Another critical limitation in deep oxidation processes under neutral conditions lies in the accumulation and re‐adsorption efficiency of intermediate products. Addressing this challenge, Tao et al. designed a composite catalyst featuring ultra‐dense, monodispersed Ru oxide clusters.^[70]^ The microscopic channels formed between these clusters facilitated the re‐adsorption and continuous oxidation of key intermediates (e.g., FFCA). Their developed two‐stage oxidation process (HMF → FFCA at room temperature, followed by FFCA → FDCA at 60°C) successfully enhanced the FDCA yield to 92.1% in neutral media while significantly suppressing polymerization side reactions.

These studies collectively demonstrate that increasing active site density/distribution, enhancing the supply and utilization efficiency of interfacial oxygen‐active species (e.g., *OH), and optimizing electrolysis methods (e.g., pulsed electrolysis) represent key strategies for achieving highly efficient FDCA synthesis in near‐neutral systems.

### 3.3 Acidic media

In acidic media (pH ≤ 5), HMF electrooxidation primarily follows the HMF‐DFF‐FFCA‐FDCA pathway, yet encounters significant bottlenecks in the aldehyde group oxidation steps (DFF‐FFCA‐FDCA). Specifically, the acidic environment not only increases the activation energy barrier for aldehyde group oxidation but also markedly suppresses the formation and stabilization of high‐valent metal oxide active species. Furthermore, strongly acidic conditions tend to promote furan ring hydrolysis and subsequent ring‐opening reactions, generating MA as a major byproduct. These limitations render the achievement of high FDCA yields in acidic systems substantially more challenging compared to alkaline and near‐neutral systems, resulting in extremely scarce research on relevant electrocatalyst design for such conditions.

Early research on HMFOR in acidic media primarily focused on noble metal‐based catalysts. In 2017, Cao et al. systematically evaluated the performance of Pt‐based alloy catalysts in an acidic solution containing 100 mM HMF and 100 mM H_2_SO_4_.^[80]^ The results demonstrated that PtRu(1:1) alloy exhibited superior catalytic performance, achieving 25% HMF conversion and 89% DFF selectivity after 17 h of reaction at 50°C, with a DFF yield (22%) more than double that of the pure Pt catalyst. The study observed further oxidation of DFF to FFCA, while pure Ru catalyst showed significantly reduced activity, likely due to the formation of inactive metal oxide species on its surface. The enhanced activity of PtRu(1:1) was attributed to alloy formation, smaller nanoparticle size, and weaker adsorption of carbonyl species on its surface. These findings provided initial insights into the potential of alloying strategies for optimizing HMFOR catalyst performance in acidic media.

With the deepening of research, developing catalysts capable of selectively oxidizing HMF to FDCA in acidic media has become an important direction, which can avoid the cumbersome pH adjustment and salt byproduct treatment required by traditional alkaline processes. In 2018, Kubota et al. first reported the direct electrooxidation of HMF using a manganese oxide (MnO_*x*_) anode in pH 1 H_2_SO_4_ solution, achieving a 53.8% FDCA yield.^[6]^ They utilized the low solubility of FDCA in strong acids to enable its spontaneous precipitation and separation from the reaction mixture, pioneering a novel “reaction‐separation integration” approach. Additionally, they observed a concurrent pathway for HMF conversion to MA under these conditions. In 2020, Gao et al. designed a TiO_*x*_@MnO_*x*_ porous nanowire electrocatalyst, leveraging the specific activity of MnO_*x*_ for HMF oxidation and the acid resistance of TiO_*x*_ nanowires, achieving approximately 24% FDCA yield in strongly acidic media.^[81]^ By 2022, Wang et al. demonstrated that their designed high‐surface‐area mesoporous δ‐MnO_2_ could also effectively catalyze HMF oxidation to FDCA and MA as value‐added products under acidic conditions, with product distribution being potential‐dependent.^[82]^ Beyond these Mn‐based catalysts, Samanta et al. reported in 2023 that a silver/silver oxide catalyst (Ag/AgO_*x*_–CN_*x*_) appeared capable of catalyzing HMF conversion to FDCA in acidic media.^[83]^

Recent studies have focused on exploring novel catalyst systems, such as molecular catalysts. In 2024, Bühler et al. reported a strategy for immobilizing molecular ruthenium complexes on mesoporous indium tin oxide electrodes via phosphonic acid anchoring groups for acidic HMFOR.^[84]^ This surface‐immobilized molecular catalyst achieved the highest reported FDCA yield (85%) and FE (91%) in acidic systems within a specially designed small‐scale reactor. This work represents a breakthrough in developing highly active and selective molecular catalysts for acidic HMFOR. However, the performance may be constrained by the specific reactor configuration, and the current density as well as long‐term stability still fall far short of practical application requirements.

## 4 REACTOR DESIGN

This chapter systematically investigates reactor design considerations for the HMFOR. While reactor concepts for HMFOR in near‐neutral/acidic systems hold long‐term promise, their development is still at an early stage, currently constrained by the low current densities achievable with existing catalysts. Given that alkaline systems are the only platform currently enabling operation at industrially relevant scales and current densities, it is both practical and instructive to focus our discussion on their reactor design. This chapter will therefore cover key aspects such as reactor configuration selection and the core engineering challenges associated with scale‐up implementation.

Achieving efficient and scalable conversion during high‐concentration HMF electrooxidation in alkaline media is significantly hindered by the inherent instability of the substrate and intermediates, leading to severe non‐Faradaic degradation (carbon loss). Notably addressing this core challenge, our group has innovatively designed and constructed a single‐pass continuous flow reactor (SPCFR) system.^[62]^ The key design strategy of this system involves: (1) adopting a high electrode area/electrolyte volume ratio to accelerate the electrochemical reaction rate; (2) significantly shortening the residence time of the substrate within the reactor to minimize its degradation; and (3) implementing a split‐flow feed of the substrate and the alkaline solution, thereby fundamentally suppressing non‐Faradaic degradation processes. By constructing an SPCFR system based on a nine‐layer stacked module, we successfully achieved efficient electrocatalytic conversion of HMF to FDCA under high‐concentration conditions, realizing a single‐pass conversion efficiency of 95.8%, FDCA selectivity of 96.9%, and FDCA concentration of 556.9 mM. More importantly, the system demonstrated robust scalability, enabling the continuous production of FDCA on a kilogram scale (1.17 kg) from HMF, thereby fully validating the significant potential of this reactor design in suppressing degradation, enhancing conversion efficiency, and enabling the large‐scale electrocatalytic upgrading of biomass.

Beyond the aforementioned continuous flow reactor design, utilizing anion exchange membrane (AEM) electrolyzers for HMFOR represents another crucial device strategy for continuous FDCA production. For instance, Ding et al. developed a rapid preparation technique for large‐area (100 cm^2^) NiCu‐based catalysts suitable for AEM electrolyzers.^[63]^ In a 25 cm^2^ single‐pass AEM electrolyzer using a 200 mM HMF electrolyte, they achieved a single‐pass FDCA yield of ≥95.0% and a selectivity of ≥99.9%. After stable operation at 10 A for 100 h, this system cumulatively produced 207.28 g of high‐purity FDCA (>99%), showcasing the promising capability of AEM electrolyzers for long‐duration continuous operation and the scaled application of catalysts. In addition, Latsuzbaia et al. used 15 wt.% HMF in 0.1 M Na_2_SO_4_ and maintained pH 12 via continuous NaOH addition during electrolysis.^[100]^ This approach in a circulated AEM‐based flow reactor suppressed HMF degradation and achieved a high FDCA production rate (∼30 g h^−1^, 3 wt.%). However, the low electrolyte pH limited the current density (<25 mA cm^−2^), increasing electrolyzer costs. Continuous NaOH addition also diluted the FDCA product, raising separation costs.

In an effort to address the challenge of scalability faced by flow systems, Chakthranont et al. developed a large‐scale single‐pass continuous stirred tank reactor (CSTR) with a 3000 cm^2^ electrode for HMF electrooxidation at 100 A.^[101]^ To suppress HMF degradation, HMF (0.15 M) and KOH (1 M) solutions were fed separately at a 2:1 ratio, reducing the retention time of HMF in base, and cooling was employed to slow degradation kinetics. This setup achieved high FDCA selectivity (98%) and production rate (75 g h^−1^). However, it also exhibited low current density (33.3 mA cm^−2^) and FDCA concentration (94.2 mM), potentially increasing capital and separation costs. Additionally, scaling up the CSTR faces technical challenges, such as addressing O_2_‐H_2_ mixing issues.

**FIGURE 6:**
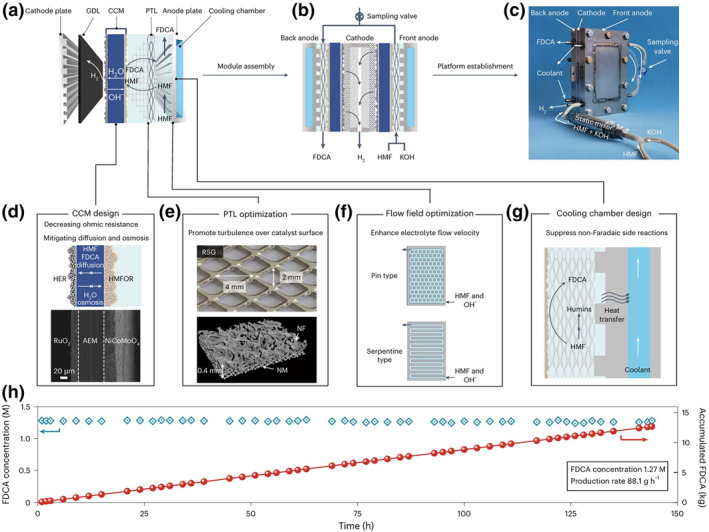
The design and performance of the SPE reactor.^[102]^ (a) An enlarged schematic of the SPE reactor configuration. It consists of a cathode for the HER and an anode for the HMFOR, an AEM‐based CCM for water and OH^−^ transport and catalyst support. GDL, gas diffusion layer. (b) A schematic of a single‐module SPE reactor. (c) A photograph of the single‐module SPE reactor connected to a static mixer. (d) A SEM image of a cross‐section of the CCM. It consists of commercial RuO_2_ as the HER catalyst and NiCoMoO_4_ as the HMFOR catalyst, with an AEM as the SPE. (e) Photographs of the PTL. (f) An illustration of the pin‐ and serpentine‐type flow field for the anode. (g) An illustration of the cooling chamber integrated into the anode plate. (h) Continuous operation of the optimized 200 cm^2^ SPE reactor at 100 A (corresponding to 0.5 A cm^−2^) for the HMFOR. AEM, anion exchange membrane; CCM, catalyst‐coated membrane; HER, hydrogen evolution reaction; HMFOR, 5‐hydroxymethylfurfural oxidation reaction; PTL, porous transport layer; SPE, solid‐state polymer electrolyte.

Recently, our group reported a solid‐state polymer electrolyte (SPE) reactor that achieves high single‐module power (654 W), industrial‐level current density (1.5 A cm^−2^), and high concentration for continuous electrocatalytic oxidation of HMF to FDCA (∼1.27 M), coupled with cathodic hydrogen production, through reactor engineering and system design that enhances convective mass/heat transfer while suppressing transmembrane permeation and diffusion (Figure 6).^[102]^ Furthermore, by modular system integration, we constructed the first kilowatt‐scale electrochemical platform, achieving an operational power of 4.3 kW at 1.0 A cm^−2^ current density with an FDCA production rate of 1.38 kg h^−1^ (∼33 kg day^−1^), marking the pilot‐scale advancement of integrated water electrolysis for hydrogen production coupled with catalytic oxidation systems.

These advancements in reactor design (namely the SPCFR, AEM electrolyzer, CSTR and SPE reactor) provide pivotal technical pathways towards overcoming the degradation issues in alkaline HMFOR, enhancing reaction efficiency, and ultimately advancing its industrial application.

## 5 CONCLUSIONS AND PERSPECTIVES

This review systematically investigates the pronounced pH‐dependent behavior of HMFOR across diverse media ranging from alkaline to acidic conditions, providing an in‐depth analysis of the mechanistic variations and corresponding catalyst/reactor design strategies. In strongly alkaline systems, abundant OH^−^ species facilitate preferential aldehyde group oxidation and intermediate deprotonation, promoting highly selective FDCA formation, though accompanied by carbon loss due to HMF self‐degradation. Under non‐strongly alkaline conditions, particularly with decreasing pH, the hydroxymethyl oxidation pathway becomes increasingly dominant, leading to complex reaction routes and incomplete oxidation that generates diverse products (such as HMFCA, DFF, and FFCA), making selectivity control a critical challenge. The corresponding catalyst design strategies exhibit distinct pH‐dependent characteristics. Alkaline systems have been extensively studied, yielding various effective approaches including SACs, elemental doping, defect engineering, interface engineering, crystal structure engineering, and microenvironment modulation. For near‐neutral systems, research priorities should focus on enhancing active site density and distribution, utilizing spatial confinement effects, strengthening interfacial active oxygen species supply, and optimizing electrolysis strategies (such as precise potential selection and electrolysis mode control). Acidic systems remain in the early exploratory stage, where manganese‐based catalysts show potential but require overcoming the significant obstacle of extremely sluggish reaction kinetics. In addition, significant breakthroughs have been achieved in reactor design for alkaline systems, particularly with the emergence of the first kilowatt‐scale SPE reactor, which has charted a course for the industrial‐scale production of HMFOR.

In the extensively studied alkaline HMFOR systems, the primary advantages lie in their fast reaction kinetics and typically high FDCA yields exceeding 90%. However, fundamental challenges remain unresolved, including severe HMF degradation losses in concentrated alkaline environments, energy‐intensive product separation and purification processes (requiring FDCA acidification and precipitation), and the resulting economic viability concerns for overall process implementation. Future strategies should not only focus on further catalyst optimization to enhance reaction rates but also prioritize reactor configuration innovations and process coupling designs. For instance, developing integrated reaction systems capable of in situ product separation or efficient electrolyte composition management could simplify purification steps and reduce energy consumption.

To further assess the industrial feasibility, key metrics such as catalyst cost, energy consumption for separation, and long‐term stability under high HMF concentrations (>500 mM) must be systematically evaluated. For example, the “oxidation‐acid precipitation” approach in alkaline media entails significant energy input for acidification and FDCA recovery, whereas acidic systems allow direct precipitation but face kinetic limitations. Quantitative comparison of separation energy, along with studies on catalyst degradation mechanisms (e.g., metal leaching under industrial conditions), will provide critical guidance for scaling up.

Near‐neutral HMFOR systems exhibit distinctive application prospects owing to their mild reaction conditions, which not only alleviate corrosion issues but also offer opportunities for targeted synthesis of various high‐value oxidation products (DFF, HMFCA, FFCA, and FDCA) through their inherent product diversity. Nevertheless, this system faces significant limitations, including substantially slower reaction rates compared to alkaline environments and difficulties in precisely regulating target product selectivity due to intricate reaction pathways. Overcoming these challenges necessitates continuous innovation in catalyst design, focusing on developing material systems with ultrahigh intrinsic activity, optimized active site distribution, and efficient water molecule activation or rapid interfacial oxygen transfer capabilities. Furthermore, advanced operational strategies such as pulsed electrolysis and gradient potential techniques should be thoroughly investigated to synergistically enhance both reaction efficiency and selectivity.

A critical consideration for practical application is balancing high activity in (weakly) alkaline media with simplified separation in neutral/acidic media. While alkaline conditions favor reaction kinetics, they complicate downstream processing. Developing adaptive systems that operate under mildly alkaline conditions during reaction and shift to neutral/acidic pH for FDCA precipitation could help reconcile separation costs with production efficiency. Such integrated process design should be explored to optimize the trade‐off between reactivity and operability.

Current research on acidic HMFOR systems remains relatively underexplored. The most significant potential advantage lies in the notable simplification of product separation, particularly for FDCA, which can be directly isolated by leveraging its low solubility in acidic solutions without additional acidification steps. This approach avoids the formation of solid salt byproducts and facilitates electrolyte recycling. However, this system faces severe practical challenges, primarily including extremely sluggish HMFOR reaction kinetics under acidic conditions, severely insufficient catalyst activity, and poor stability. Integrating noble metal (such as Ru or Ir oxides) with high acidic OER activity and promising Mn‐based catalysts to construct synergistic catalytic systems or composite interface structures may represent one crucial direction for overcoming the core bottlenecks of this system.

From a broader system integration perspective, cross‐pH adaptive catalysts capable of maintaining high HMFOR activity and stability over a wide pH range represent a promising research direction. Future studies should focus on designing robust catalytic materials—for instance through surface modification, core‐shell structuring, alloying, or multicomponent doping—that resist leaching and degradation under pH fluctuations. Research on such cross‐pH systems would not only expand the operational window of HMF electrolysis but also provide deeper mechanistic insights into the pH‐dependent behavior of catalysts and reaction pathways, thereby accelerating the development of versatile industrially relevant electrocatalytic processes.

While our review has focused on the pH‐dependent reaction mechanisms and catalyst design for enhanced activity and selectivity, it is imperative to expand the discussion to encompass the long‐term stability of electrocatalysts and the understanding of their deactivation mechanisms under operational conditions, which represent a critical frontier for the industrial translation of HMFOR. Future research should systematically investigate and contrast the deactivation pathways prevalent in different pH environments. For example, in alkaline media, catalyst degradation may stem from metal leaching, oxidative phase transformation, or fouling by reaction intermediates. In contrast, acidic conditions pose severe challenges of catalyst dissolution and support corrosion. For near‐neutral systems, salt deposition and pH drift during prolonged operation could lead to instability. Therefore, advancing the field requires a dedicated focus on developing in situ/operando characterization techniques to monitor catalyst evolution in real‐time, elucidating the root causes of deactivation. Concurrently, exploring regeneration strategies (e.g., electrochemical redox cycles, thermal treatment) and, more importantly, designing inherently robust catalysts—through strategies such as strengthening metal‐support interactions, employing protective overcoats, or using corrosion‐resistant supports—are imperative. Establishing standardized protocols for stability assessment (e.g., long‐term testing at industrial current densities >500 mA cm^−2^) will enable meaningful comparison and accelerate the development of commercially viable HMFOR processes.

Looking ahead, development prospects vary across pH systems with distinct priorities. Alkaline systems focus primarily on achieving high‐efficiency and high‐selectivity FDCA production, representing the closest to industrial application. Research emphasis will shift from catalysts to reactor optimization and improve the overall process economics. Near‐neutral systems leverage their mild conditions and inherent flexibility in product profiles. With breakthroughs in catalysts and electrolysis strategies, they hold promise as a platform technology for on‐demand production of diverse oxidation products. Acidic systems urgently require fundamental innovations in novel catalyst design, particularly in exploring highly active systems such as noble metal‐Mn‐based synergistic catalysts. If efficient acidic HMFOR catalysts can be successfully developed, the inherent advantages of simplified separation in this system would offer the most attractive solution for constructing low‐energy and low‐waste HMF electrooxidation processes.

Beyond coupling with the cathodic HER, the integration of anodic HMFOR with alternative cathodic reactions, such as CO_2_ reduction reaction (CO_2_RR), also presents a valuable route toward carbon‐neutral electrosynthesis. Systems integrating HMFOR with CO_2_RR not only improve overall energy efficiency but also produce two valuable products simultaneously (e.g., FDCA and C_2+_ chemicals), enhancing process economics. For industrial adoption, such coupled systems should be evaluated in terms of compatibility in operating conditions, long‐term stability of both electrodes, and the techno‐economic feasibility of co‐product separation and purification.

## CONFLICT OF INTEREST STATEMENT

The authors declare no conflicts of interest.
